# Supplementary material for: Adhesion pilus retraction powers twitching motility in the thermoacidophilic crenarchaeon Sulfolobus acidocaldarius
Source: Nat Commun. 2024 Jun 14;15:5051. doi: 10.1038/s41467-024-49101-7 (PMC11178785; doi:10.1038/s41467-024-49101-7)
Supplement: Supplementary file 1 — Supplementary Information [file 41467_2024_49101_MOESM1_ESM.pdf]

Supplementary Information for Charles-Orszag *et al.*, 2024

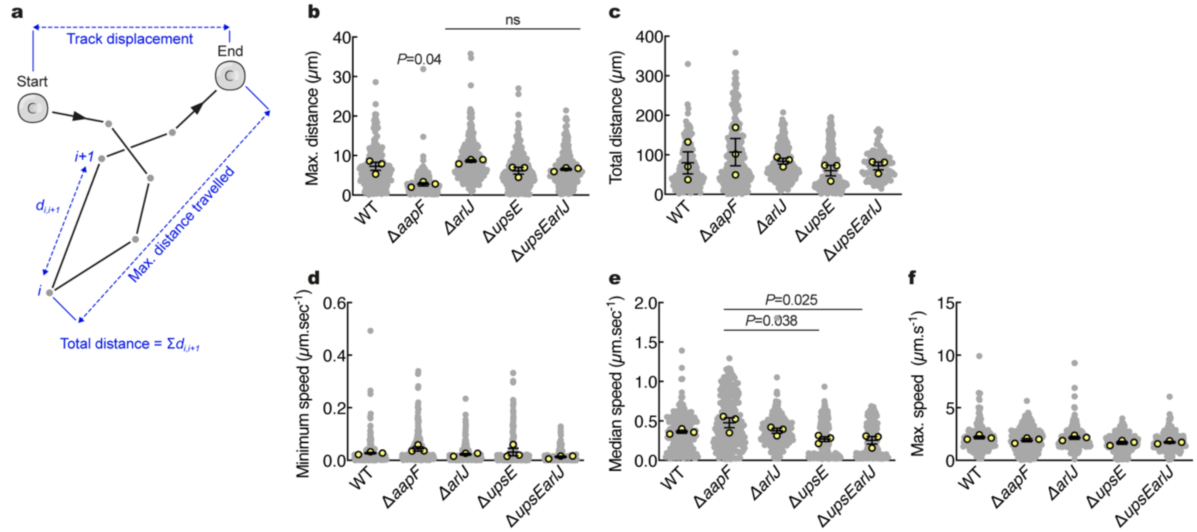

Supplementary Figure 1. Automated tracking of type IV pili mutants in *S. acidocaldarius* at high temperature (additional data set). a. Diagram representing a typical track obtained from a cell trajectory in TrackMate 7, and illustrating the biological parameters that were measured in the present study. Adapted from https://imagej.net/plugins/trackmate/algorithms. **b.** Maximum distance travelled over five minute for each indicated strain. **c.** Total distance travelled over five minutes for each indicated strain. **d-f.** Minimum, median and maximum track speeds in each indicated strain. Grey scatter dot plots correspond to the total number of cells analyzed (200 WT, 222 Δ*aapF*, 284 Δ*arlJ*, 171 Δ*upsE*, 245 Δ*upsE*Δ*arlJ)*. superimposed yellow circles represent average values from each of n=3 independent experiments and error bars represent the mean ± SEM of those three biological replicates. *P* values were calculated on the replicate means using a one-way ANOVA test with a Tukey's correction for multiple comparisons with a single pooled variance.

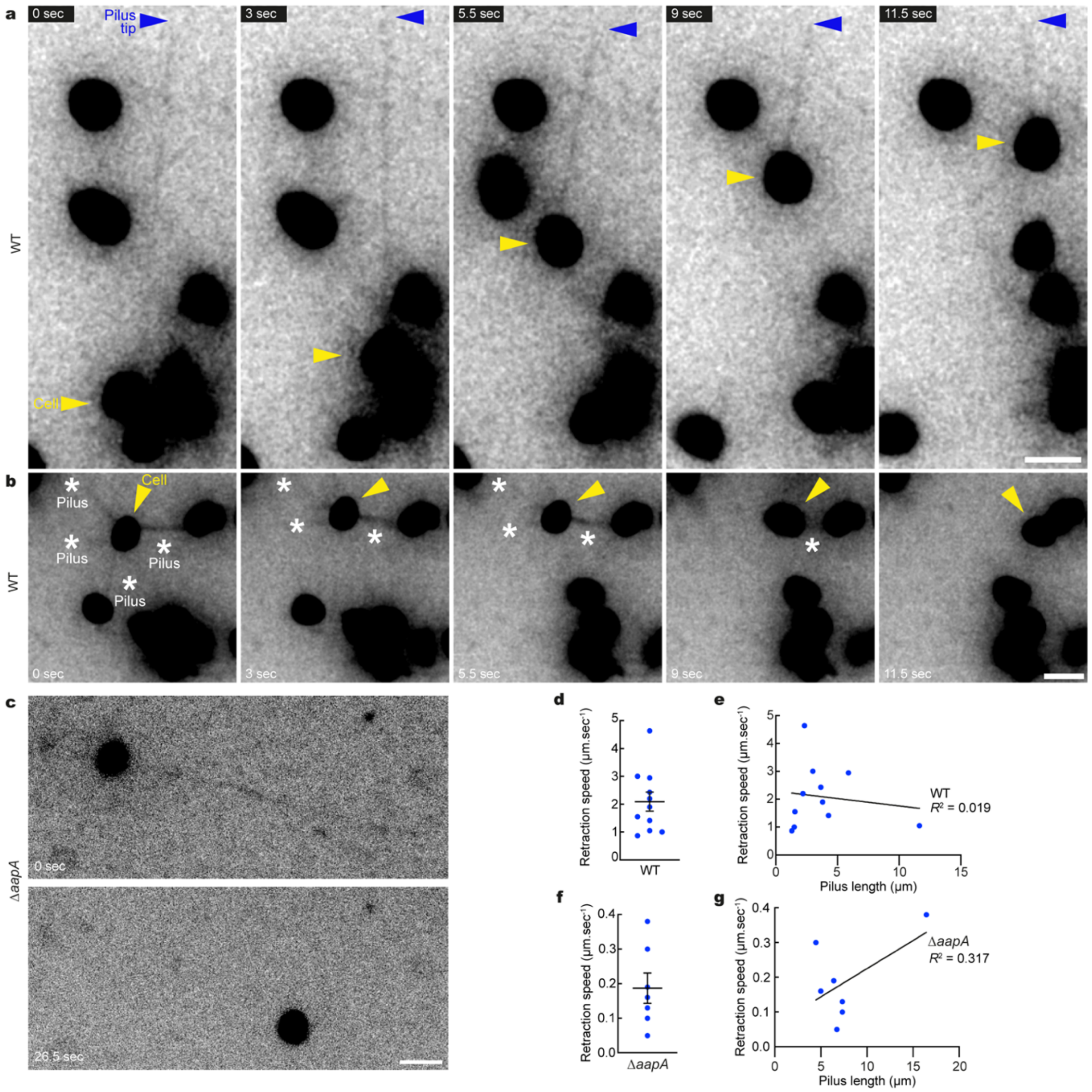

Supplementary Figure 2. Super-resolution fluorescence imaging of *S. acidocaldarius* adhesion pili dynamics at high temperature (additional data set). Surface proteins of *S. acidocaldarius* cells were labeled non-specifically with AlexaFluor 568 NHS-ester. Glass-adhered cells were imaged in Structured Illumination Microscopy (iSIM) every 500 ms. Shown are selected stills from representative movies. **a.** A WT cell (yellow arrowhead) with a pilus of 11 um (pilus tip denoted by blue arrowhead) moves across the surface as the pilus gets shorter. **b.** Pilus retraction mediate dynamic cell-cell interactions in WT. **c.** Pilus retraction observed in a deletion mutant strain for the minor pilin AapA. **d.** Mean retraction speed in single-pilus retraction events ± SEM in 11 WT cells. **e.** Retraction speed is not correlated with pilus length. **f.** Mean retraction speed in single-pilus retraction events ± SEM in 7 Δ*aapA* cells. **g.** Retraction speed is not correlated with pilus length. Scale bars, 2 µm.

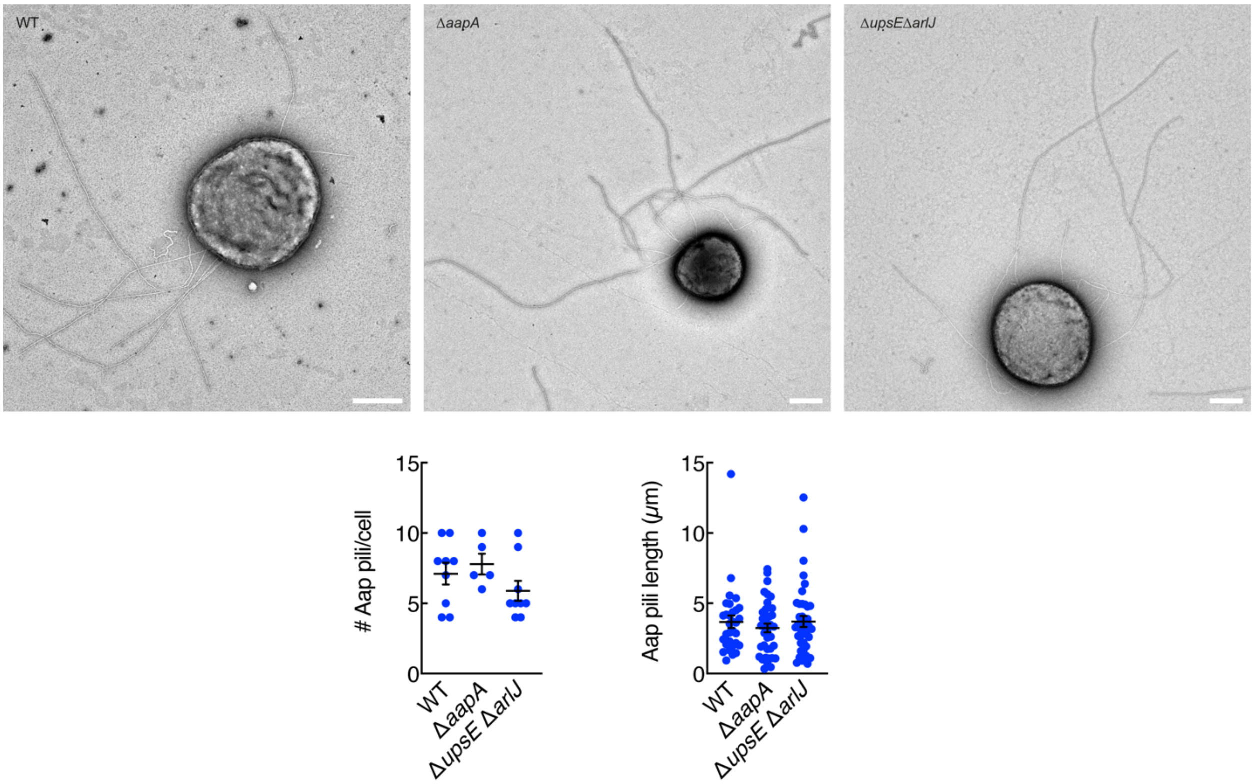

Supplementary Figure 3. Transmission electron microscopy of *Sulfolobus acidocaldarius* Aap pili. WT, Δ*aapA* and Δ*upsE*Δ*arlJ* cells were grown to exponential phase, negatively stained with 0.5% uranyl formate and imaged in transmission electron microscopy. Aap pili number and length was measured in 5-10 cells. Micrographs are representative of n=2 experiments. Scale bars, 500 nm.
