# Supplementary material for: Adhesion pilus retraction powers twitching motility in the thermoacidophilic crenarchaeon Sulfolobus acidocaldarius
Source: Nat Commun. 2024 Jun 14;15:5051. doi: 10.1038/s41467-024-49101-7 (PMC11178785; doi:10.1038/s41467-024-49101-7)
Supplement: Supplementary file 3 — Description of Additional Supplementary Files [file 41467_2024_49101_MOESM3_ESM.pdf]

Description of Additional Supplementary Files

Supplementary Movie 1. Automated tracking of type IV pili mutants in *S. acidocaldarius* at high temperature. Differential interference contrast (DIC) live-cell imaging of indicated *S. acidocaldarius* strains at 75°C. The timelapse shown is representative of n=3 independent experiments. Tracks of each cell are overlaid in a different color.

Supplementary Movie 2. Adhesion pili retraction mediate twitching motility in *S. acidocaldarius*. Surface proteins of *ΔupsEΔarlJ S. acidocaldarius* cells that only express Aap pili were labeled non-specifically with Alexa Fluor 568 NHS-ester. Glass-adhered cells were imaged in instant Structured Illumination Microscopy (iSIM) every 500 ms. The timelapse shown is representative of n=3 independent experiments.

Supplementary Movie 3. Adhesion pili retraction support dynamic cell-cell interactions in *S. acidocaldarius*. Surface proteins of *ΔupsEΔarlJ S. acidocaldarius* cells that only express Aap pili were labeled non-specifically with Alexa Fluor 568 NHS-ester. Glass-adhered cells were imaged in instant Structured Illumination Microscopy (iSIM) every 500 ms. The timelapse shown is representative of n=3 independent experiments.

Supplementary Movie 4. Single-adhesion pilus retraction event in *S. acidocaldarius*. Surface proteins of *ΔupsEΔarlJ S. acidocaldarius* cells that only express Aap pili were labeled non-specifically with Alexa Fluor 568 NHS-ester. Glass-adhered cells were imaged in instant Structured Illumination Microscopy (iSIM) every 500 ms. Single-adhesion pilus retraction events were used to measure adhesion pilus retraction speed in *S. acidocaldarius*. The timelapse shown is representative of n=3 independent experiments.

Supplementary Movie 5. Type IV pili dynamics in WT *S. acidocaldarius*. Surface proteins of WT *S. acidocaldarius* cells were labeled non-specifically with Alexa Fluor 568 NHS-ester. Glass-adhered cells were imaged in instant Structured Illumination Microscopy (iSIM) every 500 ms. The timelapse shown is representative of n=3 independent experiments.

Supplementary Movie 6. Automated tracking of *S. acidocaldarius* Aap mutants. Differential interference contrast (DIC) live-cell imaging of indicated species at 75°C. Tracks of each cell are overlaid in a different color. The timelapses shown is representative of n=3 independent experiments.

Supplementary Movie 7. Adhesion pili dynamics in Δ*aapA S. acidocaldarius*. Surface proteins of Δ*aapA S. acidocaldarius* cells were labeled non-specifically with Alexa Fluor 568 NHS-ester. Glass-adhered cells were imaged in instant Structured Illumination Microscopy (iSIM) every 500 ms. The timelapse shown is representative of n=3 independent experiments.

Supplementary Movie 8. Automated tracking of different *Sulfolobales* species at high temperature. Differential interference contrast (DIC) live-cell imaging of indicated species at 75°C. Tracks of each

cell are overlaid in a different color. The timelapses shown are representative of multiple fields of view in n=1 independent experiments.

Supplementary Movie 9. Type IV pili retraction mediate twitching motility in *Sa. solfataricus*. Surface proteins of WT *Saccharolobus solfataricus* cells were labeled non-specifically with Alexa Fluor 568 NHS-ester. Glass-adhered cells were imaged in instant Structured Illumination Microscopy (iSIM) every 500 ms. The timelapse shown is representative of n=3 independent experiments.

Supplementary Movie 10. Type IV pili retraction support dynamic cell-cell interactions in *Sa. solfataricus*. Surface proteins of WT *Saccharolobus solfataricus* cells were labeled non-specifically with Alexa Fluor 568 NHS-ester. Glass-adhered cells were imaged in instant Structured Illumination Microscopy (iSIM) every 500 ms. The timelapse shown is representative of n=3 independent experiments.
